# Letter from S. P. Hullihen, M. D.

**Published:** 1847-10

**Authors:** S. P. Hullihen


Wheeling, September 17, 1847.

Dr. Taylor:

*Dear Sir*—I was glad to hear of the harmonious action of the late meeting of the "Mississippi Valley Association of Dental Surgeons," and of the very important measures they decided upon; particularly the publication of the "Dental Register of the West." I am, however, extremely sorry that I was chosen one of its editors; although it is a situation I should be proud occupatoy, nd still more proud if I could attend to the duties, and thereby make myself useful to the profession; but suffering as I am from a nervous affection of a very anomalous character, which is always aggravated by much mental exercise or anxiety, I am admonished that it would be doing myself great injustice to add another to the many onerous engagements already pressing on me. Though I shall ever esteem it a very great privilege to be allowed to contribute to the pages of the Dental Register; yet, at the same time, I must respectfully and emphatically beg leave to decline being one of its editors.

I am, dear Sir,

Very respectfully,

Yours, &c.,

S. P. HULLIHEN.

The publication of the above letter is due from our friend, Dr. Hullihen. We are sure the Society will regret the cause which compels the Dr. to decline the duty assigned him. We had hoped to have been materially assisted by his good counsel and ready pen in this, as we think, very important enterprise—important because we view every measure which contributes to the diffusion of correct principles as such.

It affords us pleasure, however, to know, that although the Doctor declines serving as one of the Editors for the Register, yet his heart and pen will be with us, and we hope his health may soon be restored, so that our future numbers will be enriched from the results of his observation and practice.

In connection with this, we would remark, that owing to the absence of our colleague, at St. Louis, our letter informing him of his election as one of the Editors, was not received in time for preparation and transmission of any manuscript, before it was thought advisable the Register should go to press. It will afford the members of the Society some pleasure to know that he will aid us in the work, and takes much interest in so desirable an enterprise.—CIN. ED.

